# Association between *IL17* Polymorphisms and Risk of Cervical Cancer in Chinese Women

**DOI:** 10.1155/2012/258293

**Published:** 2012-09-25

**Authors:** Yi Quan, Bin Zhou, Yanyun Wang, Ruiqi Duan, Kana Wang, Qianqian Gao, Shaoqing Shi, Yaping Song, Lin Zhang, Mingrong Xi

**Affiliations:** ^1^Department of Obstetrics and Gynecology, West China Second University Hospital, Sichuan University, Chengdu 610041, China; ^2^Laboratory of Molecular Translational Medicine, West China Institute of Women and Children's Health, Key Laboratory of Obstetric & Gynecologic and Pediatric Diseases and Birth Defects of Ministry of Education, West China Second University Hospital, Sichuan University, Chengdu 610041, China

## Abstract

Interleukin-17 (IL-17) is a proinflammatory cytokine that is associated with inflammation, autoimmune disorders, and even tumors. Previous studies revealed that a large group of human malignant tumors have abnormally high IL-17 expression. In the present study, we analyzed two single-nucleotide polymorphisms (SNPs) in the *IL17A* (rs2275913) and *IL17F* (rs763780) in 311 cervical cancer patients and 463 controls using TaqMan assays. Our results indicated that the frequencies of AA genotype and A allele of rs2275913 were significantly different between the cervical cancer patients and controls (*P* = 0.008, OR = 1.32, 95% CI, 1.07–1.62). Stratified analyses revealed that the polymorphism of rs2275913 was also associated with positive peritumor intravascular cancer emboli and high clinical stage. The genotype and allele frequencies of rs763780 did not show any difference between patients and controls or relate to patient clinical characteristics. Collectively, these findings suggested that *IL17* gene polymorphism rs2275913 was associated with the susceptibility as well as positive peritumor intravascular cancer emboli and high clinical stage of cervical cancer in Chinese women.

## 1. Introduction

Cervical cancer, the second most common cancer among women worldwide, is a leading cause of cancer death in Chinese women. Each year, nearly 500,000 women develop this disease worldwide and about 80% of the cases occur in the developing country [1]. Cervical cancer is generally thought a multifactor disease. Human papillomavirus (HPV) infection has been established as the main cause of cervical cancer. Previous studies have suggested that the susceptibility to HPV can be affected by the polymorphisms in genes involved in immune response; thus, the polymorphisms in genes may play a crucial role in the pathogenesis of cervical cancer [2]. Further, accumulating researches reported that polymorphisms of a series of genes, including interleukin (IL)-1, IL-6, and IL-12 [3–5], were associated with the susceptibility to cervical cancer. Collectively, these findings strongly suggest that genetic polymorphisms may influence susceptibility to cervical cancer.

IL-17 is a relatively novel cytokine family, which plays important role in innate and adaptive immune systems. IL-17 consisted of six members (IL-17A-F), and five receptors (IL-17RA-RD and SEF) have been identified [6, 7]. IL-17A and IL-17F are reported to be secreted by Th17 cells, a distinct lineage of CD4^+^ effector cells [8]. IL-17 acts as a proinflammatory cytokine that can induce the release of certain cytokines, chemokines, matrix metalloproteinases (MMPs), and antimicrobial peptides from mesenchymal and myeloid cells. Increasing evidence has indicated that inflammation affects the microenvironment around tumors, which involves in the proliferation, migration, and survival of cancer cells [9]. Several studies have found high expression of IL-17 in various tumor tissues, including multiple myeloma, ovarian cancers, gastric cancer, and breast cancer [10–13]. Meanwhile, Zhang et al. reported that patients with cervical cancer do not only have significantly higher Th17 cell population but also higher IL-17 than normal controls [14]. *In vivo* studies in murine models also suggest that IL-17 promotes tumorigenicity of human cervical tumors [15].

Although underlying mechanism is still not quite clear, studies showed that genetic polymorphisms of *IL17* were associated with the susceptibility to a range of inflammation-related diseases, including rheumatoid arthritis, ulcerative colitis, gastric cancer, and breast cancer [16–19]. However, the role of *IL17* polymorphisms participating in the oncogenesis of cervical carcinoma remains unknown. In this study, we aimed to determine the association between the polymorphisms of *IL17* (*IL17A *and *IL17F*) and the risk of cervical cancer in Chinese women.

## 2. Materials and Methods

### 2.1. Subjects

This case-control study enrolled 311 unrelated Chinese female patients with cervical squamous cell carcinoma (mean ± SD, 41.78 ± 8.56). They were hospitalized in the West China Second University Hospital of Sichuan University, between July 2008 and May 2011. The clinical diagnosis was confirmed by histological examination of biopsy or resected tissues. Patients with cervical adenocarcinoma were excluded. The control group consisted of 463 healthy women (mean ± SD, 39.33 ± 10.24) from a regular gynecological examination. All subjects were Chinese Han population living in Sichuan province of Southwest China. All subjects have given written consent, and the study was approved by Ethics Committee of the West China Second University Hospital, Sichuan University.

### 2.2. TaqMan Probe Real-Time PCR

Genotyping of *IL17 *polymorphisms was analyzed using the TaqMan SNP genotyping assay (Applied Biosystems, ABI, Foster City, CA, USA) with the assay ID C_15879983_10 for rs2275913 and C_2234166_10 for rs763780, resp.). For *IL17A* rs2275913, TaqMan probe real-time PCR was conducted in a total volume of 4 *μ*L reaction mixture containing 2 *μ*L of 2 × TaqMan Universal PCR Master Mix, 0.2 *μ*L of 20 × SNP Genotyping Assay, 1.3 *μ*L DNase-free sterile water, and 10 ng genomic DNA. The thermal conditions of real-time PCR were as follows: 95°C for 10 min plus 49 cycles of 92°C for 15 s and 60°C for 1 min. For *IL17F* rs763780, TaqMan probe real-time PCR was conducted in a total volume of 5 *μ*L reaction mixture containing 2.5 *μ*L of 2 × TaqMan Universal PCR Master Mix, 0.25 *μ*L of 20 × SNP Genotyping Assay, 1.25 *μ*L DNase-free sterile water, and 10 ng genomic DNA. The thermal conditions of real-time PCR were as follows: 95°C for 10 min plus 59 cycles of 92°C for 15 s and 60°C for 1 min.

### 2.3. DNA Sequencing Analysis

About 10% of the samples were randomly selected to perform the repeated assays, and the results were 100% concordant. The genotypes were confirmed by DNA sequencing analysis (BigDye Terminator v3.1 Cycle Sequencing Kits; Applied Biosystems, Foster City, CA, USA). For SNP rs2275913, the following primers were used: forward 5′-ATTTCTGCCCTTCCCATTTT-3′ and reverse 5′-CCCAGGAGTCATCGTTGTTT-3′. For SNP rs763780, the following primers were used: forward 5′-GCAGAGCACTGGGTAAGGAG-3′ and reverse 5′-CTGCATCAATGCTCAAGGAA-3′.

### 2.4. Statistical Analysis

The genotype and allele frequencies of SNPs rs2275913 and rs763780 were calculated by direct count, and Hardy-Weinberg equilibrium was evaluated by chi-square test. Odds ratio (OR) and 95% confidence intervals (CIs) were calculated using logistic regression. Statistical analyses were performed with SPSS for Windows software package version 13.0 (SPSS Inc., Chicago, IL, USA). The differences were regarded significant if *P* value was less than 0.05. The study power was calculated using the Quanto 1.1.1 program (http://hydra.usc.edu/gxe/).

## 3. Results

### 3.1. Definition of Genotypes of SNP Loci rs2275913 (*IL-17A*) and rs763780 (*IL-17F*)

SNPs loci rs2275913 (*IL17A*) and rs763780 (*IL17F*) were analyzed using TaqMan probe real-time PCR. At the end of real-time PCR, the genotype of sample was distinguished on the basis of fluorescent dye; the allelic A probe for rs2275913 and allelic C probe for rs763780 were labeled with the fluorescent VIC dye and the others with the fluorescent FAM dye.

### 3.2. Comparison of *IL17A* and* IL17F* Polymorphisms between Patients and Controls

Polymorphisms at the two SNP loci were analyzed in 311 cervical cancer patients and 463 control subjects. Genotype distribution of these two polymorphisms in our cases and control subjects was consistent with the Hardy-Weinberg equilibrium. The genotype and allele frequencies of the two SNPs are summarized in Table 1. For SNP rs2275913, AA homozygous carriers had a significantly increased risk for cervical cancer compared with GG homozygous carriers in a codominant model (*P* = 0.008, OR = 1.72, 95% CI, 1.15–2.57). Subjects carrying AA homozygote of rs2275913 had a significantly increased risk for cervical cancer compared with that with allele G (AG/GG genotype) in a recessive model (*P* = 0.015, OR = 1.55, 95% CI, 1.09–2.20). Comparison of allelic frequency revealed a significant higher frequency of allele A in cervical cancer patient group in comparison to controls (47.3% versus 40.5%). To estimate the association between the A allele and cervical cancer, OR and 95% CI were calculated, and the results suggested that allele A is significantly associated with cervical cancer (*P* = 0.008, OR = 1.32; 95% CI, 1.07–1.62). The genotype and allele frequencies of locus rs763780 (*IL-17F*) did not show significant difference between cervical cancer patients and controls.

### 3.3. Analysis of *IL17A* and *IL17F* Polymorphisms and Clinicopathologic Features of Cervical Cancer

To determine whether the polymorphisms of the two loci were associated with certain clinicopathologic features, we performed stratified analyses for genotype distribution and allelic frequency in cervical cancer patients with different age, clinical stage, tumor differentiation, lymph node metastasis, parametrial invasion, and peritumor intravascular cancer emboli. The frequencies of genotype and allele of rs2275913 locus were significantly different between patient groups with high clinical stage and peritumor intravascular cancer emboli (Table 2). A significantly higher frequency of allele A was observed in patients with high clinical stage and positive peritumor intravascular cancer emboli. The results revealed that A allele was associated with high clinical stage (*P* = 0.022, OR = 1.46, 95% CI, 1.06–2.01) and peritumor intravascular cancer emboli (*P* = 0.006, OR = 1.57, 95% CI, 1.14–2.71). The genotype and allele frequencies of rs763780 did not relate to patient clinical characteristics (Table 3).

Collectively, these results indicated that allele A of rs2275913 locus was not only associated with the susceptibility of cervical cancer (Table 1) but also the high clinical stage and positive peritumor intravascular cancer emboli of cervical cancer.

## 4. Discussion

In the present study, we found that the rs2275913 of *IL17A* was correlated to the risk of cervical cancer. The homozygous AA genotype and the A allele of the rs2275913 were more frequent in cervical cancer patients. No significant association between rs763780 of *IL17F* gene polymorphism and risk of cervical cancer was observed.

Both* IL17A *and* IL17F* are located in 6p12. Highly similar amino acid sequence is found between IL-17A and -17F among the IL-17 family and IL-17A and -17F share similar functions in terms of their ability to induce chemokines that is crucial in neutrophil recruitment as well as activation. IL-17 can induce multiple proinflammatory mediators, including chemokines, cytokines, and metalloproteinases, from epithelial and fibroblast cells. Increasing evidence shows that IL-17 and IL-17-producing cells play important role in the pathogenesis of various diseases, even tumors [20].

The function of IL-17 is well defined in the pathogenesis of many diseases, but its role in the tumors is still under debate. Kato et al. found that in murine tumor models, IL-17 was an angiogenic factor, and it could promote tumor growth. The proangiogenic functions of IL-17 have also been reported by several studies [21–23]. Numasaki et al. indicated that the role of IL-17 in angiogenesis was achieved through the stimulation of vascular endothelial cell migration and regulation of a series of proangiogenic factors [24]. On the contrary, some studies indicated that IL-17 could slow or suppress tumor development and reinforce tumor-specific cytotoxic responses. Benchetrit et al. found that the growth of tumors was inhibited by IL-17 [25]. Another study also showed that tumor-specific antitumor immunity could be induced by the Meth-Acells transfected with the hIL-17 gene [26].

The functional influence of the rs2275913 that located in the* IL17A* promoter region on IL-17 production in peripheral blood mononuclear cells (PBMCs) is still unclear. Chen et al. investigated PBMCs from 27 healthy subjects and found that the SNP rs2275913 did not affect IL-17 expression level [27]. However, Espinoza et al. reported that the 197A allele-positive (AG/AA genotypes) PBMCs secreted more IL-17 than the 197A allele-negative (GG genotype) cells [28]. Although the impact of SNP rs2275913 on IL-17 production is under debate, a lot of studies have demonstrated that the polymorphism do associate with a wide range of human diseases, including acute graft-versus-host disease, rheumatoid arthritis, and ulcerative colitis [16, 17, 28]. There are some reports that demonstrate the influence of polymorphisms of* IL17F* in the risk for human disorders. Kawaguchi et al. reported that the *IL17F* 7488T/C (rs763780) variant, which causes a His-to-Arg substitution at aminoacid 161 (H161R), suppresses the expression and/or activity of wild-type IL-17F. In addition, it has been shown that it influences the risk of asthma [29]. The associations between SNP rs763780 of *IL17F* and several diseases, such as asthma, inflammatory bowel disease, and Crohn's disease, have also been investigated [17, 30–33]. These two SNPs have also been investigated in the gastric cancer [18] and breast cancer [19]. A study by Shibata et al. found that rs2275913 of* IL17A* was significantly associated with the development of gastric cancer [18]. In addition, it was demonstrated that rs2275913 in* IL17A* but not* IL17F* was associated with the risk of breast cancer [19]. These studies suggest that *IL17* gene may play a crucial role in the pathogenesis of tumorigenesis. However, it remains unknown whether genetic polymorphisms in *IL17A* and *IL17F* influence the risk of cervical cancer development.

Therefore, we conducted a case-control study to investigate the association between* IL17* polymorphisms and the susceptibility of cervical cancer. In the present study, the results showed that the SNP rs2275913 of *IL17A*, but not the SNP rs763780 of* IL17F* was associated with the susceptibility of cervical cancer. The frequencies of rs2275913 AA homozygote and A allele were significantly higher in cervical cancer patients than in controls. Stratified results revealed that *IL17A* polymorphism was significantly associated with positive peritumor intravascular cancer emboli and high clinical stage that are associated with the survival rate of the patients. No association was found between* IL17A* and *IL17F* polymorphisms and results of stratified analyses by other clinical characters. Although the expression of IL-17 in several tumors has been detected, including breast, prostate, gastric, and bladder cancer, its function in the context of tumors remains controversial [34]. Increasing evidences showed that IL-17 was involved in tumorigenicity of human cervical cancer. In the plasma of the cervical cancer patients, there was a significant increase in the concentration of the IL-17 [14]. Tartour et al. reported that human cervical cancer cell lines stimulated with recombinant IL-17 could upregulate IL-6 which played important role in the pathogenesis of cervical cancer and macrophage recruitment at the tumor site, and when transfected with IL-17 cDNA they show significantly higher tumour growth in athymic nude mice [15]. These results indicated the role of IL-17 in promoting tumorigenicity of human cervical cancer.

In conclusion, the current findings indicate that the polymorphism of *IL17A* may influence the susceptibility to cervical cancer in the Chinese population. The pathophysiologic features of cervical cancer may be also affected by the polymorphisms. The investigation might have some limitations. Our study is inherently limited by the study design and relatively small sample size, which weakens our ability to solidify statistic associations. Further studies in different population and with a larger size of samples are needed to identify the association between the *IL17A *and* IL17F* genes and the risk of cervical cancer.

## Figures and Tables

**Table 1:** Genotype and allele distribution of two single-nucleotide polymorphism loci in cervical squamous cell carcinoma patients and controls.

Genotype	Patients (%)	Controls (%)	*P* value	OR (95% CI)
	*N* = 311	*N* = 463		
rs2275913				
GG	93 (29.9)	168 (36.3)	Ref	
AA	76 (24.4)	80 (17.3)	**0.008**	**1.72 (1.15–2.57)**
AG	142 (45.7)	215 (46.4)	0.295	1.19 (0.86–1.67)
AA	76 (24.4)	80 (17.3)	**0.015**	**1.55 (1.09–2.20)**
GG/AG	235 (75.6)	383 (82.7)		
Allele				
A	294 (47.3)	375 (40.5)	**0.008**	**1.32 (1.07–1.62)**
G	328 (52.7)	551 (59.5)		

rs763780				
TT	222 (71.4)	332 (71.7)	Ref	
CC	4 (1.3)	5 (1.1)	0.445	0.61 (0.16–2.29)
CT	85 (27.3)	126 (27.2)	0.957	1.01 (0.73–1.39)
TT	222 (71.4)	332 (71.7)	0.922	1.02 (0.74–1.40)
CT/CC	89 (28.6)	131 (28.3)		
Allele				
C	93 (15.0)	136 (14.7)	0.886	1.02 (0.77–1.36)
T	529 (85.0)	790 (85.3)		

Values with *P* < 0.05 are shown in bold.

*N*: number; OR: odds ratio; CI: confidence interval.

**Table 2:** Analysis of patient characteristics and polymorphism of locus rs2275913.

Characteristics	Total number *N* = 311	Genotype			*P* value	Allele		*P* value	OR (95% CI)
		AA	AG	GG		A	G		
Age									
≤45 year	200	51 (25.5)	95 (47.5)	54 (27.0)	0.324	197 (49.3)	203 (50.7)	0.184	1.25 (0.90–1.74)
>45 year	111	25 (22.5)	47 (42.4)	39 (35.1)		97 (43.7)	125 (56.3)		
Clinical stage									
I	135	20 (14.8)	73 (54.1)	42 (31.1)	Ref	113 (41.9)	157 (58.1)	Ref	**1.46 (1.06–2.01)** 2.03 (0.69–6.04)
II	169	53 (31.4)	67 (39.6)	49 (29.0)	**0.002**	173 (51.2)	165 (48.8)	**0.022**	
III	7	3 (42.8)	2 (28.6)	2 (28.6)	0.198	8 (57.1)	6 (42.9)	0.193	
Tumor differentiation									
Poor	251	59 (23.5)	118 (47.0)	74 (29.5)	0.588	236 (47.0)	266 (53.0)	0.795	0.95 (0.64–1.41)
Well-moderate	60	17 (28.3)	24 (40.0)	19 (31.7)		58 (48.3)	62 (51.7)		
Lymph node status									
Positive	55	20 (36.4)	20 (36.4)	15 (27.2)	0.070	60 (54.5)	50 (45.5)	0.092	0.70 (0.46–1.06)
Negative	256	56 (21.9)	122 (47.6)	78 (30.5)		234 (45.7)	278 (54.3)		
Parametrial invasion									
Positive	95	24 (25.3)	48 (50.5)	23 (24.2)	0.328	96 (50.5)	94 (49.5)	0.280	1.21 (0.86–1.70)
Negative	216	52 (24.1)	94 (43.5)	70 (32.4)		198 (45.8)	234 (54.2)		
Peritumor intravascular cancer emboli									
Positive	121	43 (35.5)	45 (37.2)	33 (27.3)	**0.001**	131 (54.1)	111 (45.9)	**0.006**	**1.57 (1.14–2.71)**
Negative	190	33 (17.4)	97 (51.1)	60 (31.6)		163 (42.9)	217 (57.1)		

*N* corresponds to the number of individuals.

Adjusted by age, clinical stage, tumor differentiation, lymph node status, parametrial invasion, and peritumor intravascular cancer emboli.

Poor: undifferentiated cell with a spindle shape and presence of nuclear fission; moderate: individual cell keratinization but no cellular bridge or keratin pearl formation; well: differentiated cells with keratin pearl.

Boldfaced values indicate a significant difference at the 5% level.

**Table 3:** Association between the allele frequency and genotype distribution of rs763780 polymorphism and patient's characteristics.

Characteristics	Total number *N* = 311	Genotype			*P* value	Allele		*P* value	OR (95% CI)
		CC	CT	TT		C	T		
Age									
≤45 year	200	3 (1.5)	53 (26.5)	144 (72.0)	0.831	59 (14.8)	341 (85.2)	0.850	0.96 (0.61–1.51)
>45 year	111	1 (0.9)	32 (28.8)	78 (70.3)		34 (15.3)	188 (84.7)		
Clinical stage									
I	135	3 (2.2)	43 (31.9)	89 (65.9)	Ref	49 (18.1)	221 (81.9)	Ref	0.66 (0.42–1.03) 0.347 (0.04–2.72)
II	169	1 (0.6)	41 (24.3)	127 (75.1)	0.137	43 (12.7)	295 (87.3)	0.064	
III	7	0 (0.0)	1 (14.3)	6 (85.7)	0.547	1 (7.1)	13 (92.9)	0.292	
Tumor differentiation									
Poor	251	4 (1.6)	72 (28.7)	175 (69.7)	0.312	80 (15.9)	422 (84.1)	0.159	1.56 (0.84–2.91)
Well-moderate	60	0 (0.0)	13 (21.7)	47 (78.3)		13 (10.8)	107 (89.2)		
Lymph node status									
Positive	55	0 (0.0)	16 (29.1)	39 (70.9)	0.626	16 (14.5)	94 (85.5)	0.895	0.96 (0.54–1.72)
Negative	256	4 (1.6)	69 (26.9)	183 (71.5)		77 (15.0)	435 (85.0)		
Parametrial invasion									
Positive	95	2 (2.1)	28 (29.5)	65 (68.4)	0.574	32 (16.8)	158 (83.2)	0.381	1.23 (0.77–1.96)
Negative	216	2 (0.9)	57 (26.4)	157 (72.7)		61 (14.1)	371 (85.9)		
Peritumor intravascular cancer emboli									
Positive	121	2 (1.7)	28 (23.1)	91 (75.2)	0.403	32 (13.2)	210 (86.8)	0.335	0.80 (0.50–1.27)
Negative	190	2 (1.1)	57 (30.0)	131 (68.9)		61 (16.1)	319 (83.9)		

*N* corresponds to the number of individuals.

Adjusted by age, clinical stage, tumor differentiation, lymph node status, parametrial invasion and peritumor intravascular cancer emboli.

Poor: undifferentiated cell with a spindle shape and presence of nuclear fission; moderate: individual cell keratinization but no cellular bridge or keratin pearl formation; well: differentiated cells with keratin pearl.

Boldfaced values indicate a significant difference at the 5% level.
